# Security Evaluation of Arduino Projects Developed by Hobbyist IoT Programmers

**DOI:** 10.3390/s23052740

**Published:** 2023-03-02

**Authors:** Fulvio Corno, Luca Mannella

**Affiliations:** Dipartimento di Automatica e Informatica, Politecnico di Torino, Corso Duca degli Abruzzi 24, 10129 Turin, Italy

**Keywords:** Arduino, cybersecurity, Do It Yourself (DIY), GitHub, Internet of Things (IoT), hobbyist programmers, novice programmers, security analysis

## Abstract

Arduino is an open-source electronics platform based on cheap hardware and the easy-to-use software Integrated Development Environment (IDE). Nowadays, because of its open-source nature and its simple and accessible user experience, Arduino is ubiquitous and used among hobbyist and novice programmers for Do It Yourself (DIY) projects, especially in the Internet of Things (IoT) domain. Unfortunately, such diffusion comes with a price. Many developers start working on this platform without having a deep knowledge of the leading security concepts in Information and Communication Technologies (ICT). Their applications, often publicly available on GitHub (or other code-sharing platforms), can be taken as examples by other developers or downloaded and used by non-expert users, spreading these issues in other projects. For these reasons, this paper aims at understanding the current landscape by analyzing a set of open-source DIY IoT projects and looking for potential security issues. Furthermore, the paper classifies those issues according to the proper security category. This study’s results offer a deeper understanding of the security concerns in Arduino projects created by hobbyist programmers and the dangers that may be faced by those who use these projects.

## 1. Introduction

The proliferation of the Internet of Things (IoT) has led to a significant increase in the use of microcontroller-based platforms such as Arduino. Arduino is an open-source electronics platform that is based on inexpensive hardware and an easy-to-use software development environment. It has become a popular choice among novice programmers and hobbyists for building Do It Yourself (DIY) projects, especially in the IoT domain. However, the simplicity and accessibility of Arduino also mean that many developers may not have a deep understanding of the leading security concepts in the Information and Communication Technology (ICT) field.

The rise in popularity of Arduino has led to a large number of open-source projects being developed and shared on various online platforms such as GitHub [1], Arduino Project Hub [2], and Instructables [3]. These projects, often developed by hobbyist programmers, can be easily downloaded, modified, and used by non-expert users, potentially leaving them vulnerable to various types of attacks. Furthermore, as these projects are publicly available, they may serve as practical examples for other hobbyist programmers to build upon, potentially perpetuating any security issues.

This paper aims to understand the most common security issues in the most popular open-source Arduino projects developed by hobbyist programmers and to classify them according to their nature. To achieve this, a set of projects was retrieved from GitHub, one of the largest code-hosting platforms in the world [4]. In addition to its diffusion, the main advantage of using GitHub is that, compared with other similar platforms, it offers advanced research functionalities that we exploited in our research. The findings of this study provide valuable insights into the security of Arduino projects developed by hobbyist programmers and the potential risks for possible users of these projects.

The rest of this paper is structured as follows: Section 2 provides an overview of the existing related literature. Section 3 describes the methodology used to conduct the analysis. The reference classification, the research criteria, the approach used to classify the security issues found in the projects, and the list of projects are presented in detail. Section 4 presents the analysis results, focusing on each security category in detail. It shows which security issues are present in the projects according to the reference classification. Moreover, Section 5 discusses the analysis results, highlighting the implications of the findings for hobbyist programmers and users of Arduino projects. In the end, Section 6 summarizes the main findings of the paper.

## 2. Related Work

As the number of connected devices continues to increase, Internet of Things (IoT) systems’ security has been a growing concern in recent years. Indeed, according to a report published by IoT Analytics [5], despite the chip shortage that started in 2020, the IoT domain continues to grow. It is forecasted to have around 27 billion connected IoT devices in 2025.

Among this massive variety of devices, one of the most popular platforms for building IoT systems among hobbyist and novice programmers is Arduino, an open-source electronics platform based on inexpensive hardware and an easy-to-use software development environment [6]. Indeed, not only is Arduino increasingly being adopted in many courses (in both schools and universities) to introduce students to programming [7], but it is also considered a tool that can help students to grow from hobbyists to professionals [8]. Moreover, the Arduino platform has been widely adopted for its simplicity and cost-effectiveness in prototyping devices for a range of different industries, proving to be a valuable tool for academics, hobbyists, and professionals in creating functional and energy-efficient products [9]. Among them, there are also many examples of how Arduino is effective in prototyping IoT systems [10,11].

Naturally, hobbyist programmers do not always have a deep understanding of the main disciplines needed to develop good solutions in the field of Information and Communication Technology (ICT) (e.g., software engineering, performance, reliability, and usability). Among these subjects, this paper focuses on analyzing the cybersecurity aspects of the projects developed by hobbyist programmers.

Indeed, the simplicity and accessibility of Arduino increase the risk that a project could be developed without proper knowledge of the cybersecurity field. A previous study has already shown how often novice IoT developers do not consider common security issues when approaching an IoT project [12]. This lack of knowledge (or attention) has a direct impact on the developed projects, increasing the opportunities for malicious users to compromise IoT devices. Furthermore, it is noteworthy that a compromised device in a network is dangerous for itself and potentially for all the other connected devices. Indeed, some studies demonstrate how a compromised device can have an impact even on other resources in the same network [13]. Moreover, a compromised machine can also be part of a botnet [14] involved in Distributed Denial of Service attack (DDoS) such as the famous Mirai botnet [15].

For these reasons, many scholars are conducting research to understand the security situation of IoT systems [16,17]. According to their research, even if the security of IoT devices has gradually risen, there is still work to be carried out to make these systems secure. For instance, considering smart homes, an analysis conducted a few years ago on a very large sample of dwellings (16 million) discovered how widespread they are and how they are affected by well-known security vulnerabilities [18].

Discussing Arduino security specifically, other researchers have already studied whether Arduino boards and other microcontrollers adopted in the IoT domain are secure. For instance, Strobel et al. demonstrated why implementing sensitive applications on Commercial Off-the-Shelf (COTS) microcontrollers can lead to severe security problems [11]. Moreover, in another study, Alberca et al. showed how Arduino Yun—a dual-board microcontroller capable of supporting Linux distribution designed to create IoT projects—can be a victim of several attacks [19]. In addition, Audreay Ann Gendreau, in her paper “Internet of Things: Arduino Vulnerability Analysis” [20], examines several types of attacks and vulnerabilities related to the microcontroller world, with a specific focus on Arduino devices.

Despite the amount of research that has been conducted on the security of Arduino-based IoT systems, we were not able to find research specifically focused on the projects developed by hobbyist programmers. By focusing on the security issue of open-source projects developed by hobbyist developers—and classifying them according to the proper category—this paper aims to provide valuable insights that can be used to improve the security of Arduino-based IoT systems and consequently protect eventual end-users from these potential risks.

To choose the proper classification method for the observed security issues, we analyzed the approach followed by recent literature. Similar classification tasks, in the literature, were conducted mainly according to the different layers of an IoT architecture [21,22], to some particular security issues [22,23], or considering a few applications and specific use-case scenarios [24]. For our purpose, we adopted the classification proposed by Pal et al. in: “Security Requirements for the Internet of Things” [25]. Indeed, according to our knowledge, this is one of the more complete threat models explicitly proposed for the IoT domain. In Section 3.1, the paper better describes the adopted classification.

## 3. Materials and Methods

This section presents the methodology used to conduct the security analysis. Section 3.1 describes the reference classification used to classify the security issues found in the projects. Section 3.2 explains the criteria used to select the projects. Section 3.3 describes the methodology adopted to conduct the analysis. In addition, Section 3.4 highlights the novelty and contributions provided by the paper. To conclude, Section 3.5 presents those projects with a brief description of each of them.

### 3.1. Project Classification

As we already discussed in Section 2, to classify the observed security threat, we used the classification proposed by Pal et al., in “Security Requirements for the Internet of Things” [25], one of the more complete threat models explicitly proposed for the IoT domain. In their work, they address various aspects of the IoT environment and categorize security threats and attacks into five areas (which could be overlapping):

1. **Communications**:
  Attacks and threats related to the communication between the devices in wired or wireless mediums (e.g., routing channels and data transmission).
2. **Device/Services**:
  Attacks and threats related to the physical IoT devices and the associated low-level services (e.g., battery).
3. **Users**:
  Attacks and threats directed against the human beings involved in an IoT system (e.g., privacy and identity disclosure).
4. **Mobility**:
  Attacks and threats mainly related to two different scenarios: when the specific location of the device is known to the attacker and when the device is moved to a different network, e.g., location-privacy and tracking.
5. **Integration of Resources**:
  Attacks and threats that could exist in heterogeneous infrastructures. For instance, obtaining access to a resource is possible to obtain cascade access to other resources or services.

These areas (called security categories) are divided into security issues. For each security issue, the cited paper reports a non-exhaustive list of possible threats and attacks.

Inside the Communications security category, there are four security issues: *Routing* attacks, *Active* attacks, *Passive* attacks, and *Flooding*. *Routing* attacks target routing protocols and network traffic to disrupt or redirect information flow. Examples include blackhole, wormhole, and pharming attacks. In an *Active* data attack, valid data packets are targeted and altered or deleted, such as channel jamming and various forms of data tampering. *Passive* data attacks aim to gain information without altering communications, such as eavesdropping and traffic analysis. *Flooding* attacks involve introducing new packets into the network, including SYN flooding attacks, which can also be considered a Denial of Service (DoS) attack. DoS attacks are particularly concerning for IoT systems due to the resource constraints of many IoT devices, as a small amount of false traffic can compromise an IoT device [26].

Security threats in the Device/Services area can be broadly classified into four issues: *Physical Attacks*, *Device Subversion*, *Device Data Access*, and *Device Degradation*. *Physical Attacks* target the device itself and can include damage, disconnection, and destruction. *Device Subversion* attacks involve taking full or partial control over an IoT device, either individually or in large groups, and using it to cause malfunction or provide incorrect outputs. *Device Data Access* attacks involve infecting one or more IoT devices and using them to access sensitive data without the user’s knowledge. Furthermore, this kind of attack is complicated to spot because devices usually seem to work as usual [26]. To conclude, *Device Degradation* is a form of DoS attack that targets the functioning of devices to prevent access to a service. These attacks can cause memory exhaustion and battery corruption, compromising the entire system’s operations.

The security issues associated with Users are divided into four categories: *Trust*, *Data Confidentiality*, *Identity Management*, and *Behavioral Threats*. Overall, these categories of security threats related to users in the IoT highlight the importance of considering human factors in securing the IoT. *Trust*-related attacks undermine the trust relationship between devices, decreasing the system’s overall security. Among them, there are self-promotion (a malicious node provides good recommendations for itself), bad-mouthing (a malicious node provides bad recommendations against good nodes), and good-mouthing attacks (malicious nodes provide good recommendations for other compromised nodes) on other peers within the system. *Data Confidentiality* threats compromise users’ privacy and personal information. *Identity Management* threats exploit the multiple identities of users in the IoT to gain unauthorized access. *Behavioral Threats* are created by malicious or selfish behaviors of the users via social engineering attacks. Among these issues, we can also find free-rider attacks, in which the attacker benefits from the community without contributing their share to the community.

Security issues related to Mobility can be broadly categorized into three main areas: *Dynamic Topology*/Infrastructure, *Tracking and Location Privacy*, and *Multiple Jurisdictions*. *Dynamic Topology*/Infrastructure issues include challenges in routing and transmitting data due to the changing network structure and the resource-constrained nature of IoT devices. Issues related to *Tracking and Location Privacy* have the potential for breaches of personal privacy through the disclosure of a user’s geographical location and activities. Issues related to *Multiple Jurisdictions* include challenges in managing and protecting users’ data privacy due to the different regulations in different jurisdictions.

Security threats in the Integration of Resources area can be broadly classified into three categories: *Cross-Domain Administration*, *Cascading Resources*, and *Interoperability*. In *Cross-Domain Administration*, threats may arise from the cooperation and interaction of components from different network domains. This behavior could result in insecure networks and a lack of trust in network connections. *Cascading Resources* threats may occur when a low-level security breach cascades up to affect higher-level services and applications that depend on the compromised component. For example, an attacker may be able to gain access to a building by compromising a motion sensor in a home automation system. *Interoperability* threats may arise from the need for multiple systems to work together and the potential for attackers to exploit any issues in the IoT system. This behavior could result in vulnerabilities as data are moved and communicated between components, such as in a smart healthcare system where a patient’s private and sensitive information may be compromised.

### 3.2. Research Criteria

To conduct this study, we searched for IoT Arduino-based open-source projects that could be developed by a hobbyist programmer. We obtained the projects from GitHub, a leading code-hosting platform that also offers advanced search capabilities [4].

Hence, we used these keywords: “Arduino”, “DIY”, and “IoT”. In addition, for IoT, we also included the alternative spellings: “Internet of Things”, “Internet-of-Things”, and “InternetOfThings” plus the term Ubiquitous Computing written in the following different ways: “Ubi Comp”, “Ubi-Comp”, “UbiComp”, “Ubiquitous Computing”, “Ubiquitous-Computing”, and “UbiquitousComputing”. Moreover, as alternative spellings of DIY, we used “Do It Yourself”, “Do-It-Yourself”, and “DoItYourself”. Furthermore, we also considered the words “Amateur”, “Hobby”, and “Hobbyist” in place of DIY.

GitHub searches for these keywords inside its metadata. Among them, there are the repositories’ names, tags, and the “about” sections. The keyword can be present in any of these fields to obtain that repository as a result. To provide an example, a repository with “IoT” in its name, “Arduino” among its tags, and “DIY” inside its “about” will be returned (like the project described in Section 3.5.6). We mixed these keywords, obtaining 70 different queries. All these keywords were executed, and we collected all the obtained repositories. It was necessary to run many different queries because, currently, GitHub does not support queries with more than five operators (i.e., no more than five AND, OR, and NOT are allowed in a single query). The executed queries were case-insensitive.

However, a security issue in a project is more dangerous if we consider that other users could also adopt such a project. To express their interest in a specific repository, GitHub users can assign a “star” to them. Therefore, we used this rating to extract a set of projects to analyze that could be reasonably downloaded and deployed by other users. To be included in our analysis, a repository needed have a minimum of five stars. From our point of view, this value is sufficiently high to demonstrate that some users found that repository interesting (and that includes the possibility that they were also using it). Furthermore, to try to identify projects developed by hobbyist programmers, we set an upper threshold to the number of stars. Indeed, it is infrequent that a project realized by a hobbyist developer achieves a considerable number of stars. Hence, we set this upper threshold to 50 stars.

Exploring the repositories, we noticed that the keyword “Arduino” is often associated with similar and compatible microcontrollers (e.g., ESP8266 or ESP32). Considering that these microcontrollers are strongly based on the Arduino architecture and can be programmed using the Arduino Integrated Development Environment (IDE), they were also included in our analysis.

On the contrary, we excluded from the analysis the repositories without code for Arduino devices—i.e., they use the “Arduino” tag because they interact with Arduino-like devices—and repositories that are documentation or libraries (considering that hobbyist or novice developers rarely design libraries). We also excluded those projects that cannot be considered as hobbyist projects anymore. For instance, we excluded projects managed by more than three developers or with more than 3000 Single Lines of Code (SLoC). Moreover, we excluded projects that do not use English as their primary language.

To summarize, our research criteria for matching public GitHub repositories are as follows:

- Research Keywords = [Arduino && IoT && DIY]
  ○ Alternative spellings and synonyms of IoT:
    “Internet of Things”, “Internet-of-Things”, “InternetOfThings”, “Ubi Comp”, “Ubi-Comp”, “UbiComp”, “Ubiquitous Computing”, “Ubiquitous-Computing”, and “UbiquitousComputing”.
  ○ Alternative spellings and synonyms of DIY:
    “Do It Yourself”, “Do-It-Yourself”, “DoItYourself”, Amateur, “Hobby”, and “Hobbyist”.
- Stars = [5; 50]
- No libraries nor documentation
- Project must seem developed at the hobbyist level. For instance:
  ○ No more than 3 involved developers
  ○ No more than 3000 SLoC
- Written in English

In July 2021, we used the methodology previously outlined to search for relevant projects on GitHub, resulting in the acquisition of 16 repositories. In Section 3.5, each project will be briefly described. If not specified, each project is developed and maintained by only one developer and requires internet connectivity.

### 3.3. Methodology

The methodology adopted in this research activity consisted of several steps. Firstly, we selected the repositories for analysis based on the research criteria described in Section 3.2. The next step was to analyze each project for potential security issues. A cybersecurity researcher and two master’s students in computer engineering were responsible for analyzing the repositories.

Preliminarily, a set of guidelines has been defined by the paper’s authors to ensure consistency in the analysis. To provide some examples of those guidelines, for *Active* or *Passive* attacks, the research team looked for encryption mechanisms that could prevent such attacks. Indeed, without such protection, these attacks could always be feasible. For the Data Confidentiality issues, the team looked for any kind of data related to the user in the source code (e.g., personal data of the users). Instead, to consider a Cascading Resources attack feasible, the team searched for usernames, passwords, access tokens, and any information that could grant access to other resources beyond the Arduino board. Each team member repeated the analysis process independently and systematically for each security issue.

Each member of the team independently conducted a systematic manual analysis of the repositories to identify and classify the security issues. The classification of the projects’ issues was based on the categories described in Section 3.1.

After the three independent analyses were conducted, the research team discussed their findings and converged on a unique outcome. Any discrepancies in the findings were resolved through further discussion and analysis. This methodology allowed us to conduct a thorough and comprehensive analysis of the selected repositories and identify potential security issues. The use of multiple researchers also ensured the reliability and validity of the findings.

It is essential to specify that our analysis was focused on Arduino-like projects; therefore, we did not deeply investigate the security of possible included companion applications. In particular, we consider only the security issues related to the source code that an Arduino-like device could execute. Furthermore, we reviewed only the source code contained in the master/main branch without considering alternative branches.

Even if it would be interesting to evaluate even potential harm caused by each kind of attack, it is almost impossible to know in advance the whole network setup of the end users and the possible changes they made to the originally designed project. For this reason, this analysis does not evaluate the severity of a successful attack. This paper specifies whether each attack is reasonably feasible or not, independently of its risk.

Even if we conducted our analysis to the best of our knowledge, cybersecurity is a very complex subject. Therefore, we cannot guarantee that a project does not have a particular security issue, even if we did not notice any vulnerability for a specific attack related to it.

### 3.4. Novelty and Contribution

This paper provides a source-code-level analysis of the security issues of Arduino-like projects developed by hobbyist programmers. To the best of our knowledge, this is one of the first studies to provide a systematic overview of the security threats that affect such projects. By identifying and discussing the potential exploitation of these security issues, we aim to provide scholars, developers, and users with insights into the risks associated with this type of project.

In particular, one of the main contributions of the work lies in the identification of the five security categories that can affect Arduino-like projects, which are based on the classification proposed in the paper “Security Requirements for the Internet of Things: A Systematic Approach” [25]. By analyzing how developed projects could fall into one of these categories, we aim to provide a comprehensive and structured view of the security threats that can affect these projects. Another important aspect of our work is the adoption of a manual analysis of the source code of the selected repositories. This approach, together with a qualitative understanding of the projects, allowed us to identify a wide range of security issues. In addition, the involvement of three different people in the manual analysis process ensured that multiple perspectives and levels of expertise were considered, improving the reliability of our results.

Overall, we believe that our work provides a better understanding of the security challenges faced by Arduino-like projects. Highlighting these issues, this paper can help guide the development of more secure and resilient solutions.

### 3.5. Analyzed Projects

In this section, the paper presents the retrieved repositories. For each project, the following are reported: a brief description of the project’s purpose, the number of stars achieved by the repository when the project was downloaded, the approximate number of Source Lines of Code (SLoC) written for the Arduino-like board, the number of involved developers, and if the board has potentially direct access to the internet. Table 1 summarizes the main specifications of all the involved projects.

#### 3.5.1. Iotinator [27]

According to its developer, iotinator is “the DIY multipurpose home IoT solution […] an IoT application you can already use”. This project has around 2050 SLoC. It is a master model designed to manage other slave modules programmed by the same developer. On the download date, it had eight stars.

#### 3.5.2. Probee [28]

Probee is an Arduino & Raspberry Pi Open Hardware robotic self-navigating car. This project has more than 960 lines of code and is not connected to the internet. It is composed of an Arduino and a Raspberry coupled together. The Arduino is connected to a motor, a servo, a laser, a LED, an LCD display, and an ultrasonic sensor. On the download date, it had six stars.

#### 3.5.3. Arduino Commands [29]

Arduino Commands is an iOS App that allows users to control basic Arduino functionalities through Wi-Fi/Ethernet using HTTP Requests. This repository contains the code that has to be installed on the board to be governed by the app. This project has around 300 SloC. It creates a local web server that can be used for interacting with onboard components such as LED. On the download date, it had seven stars.

#### 3.5.4. TwitterMoodLight [30]

This project asserts the general mood of people parsing public tweets available on the web. The application then translates the mood into a color displayed by a lamp. Without considering the imported libraries, this project has more than 760 lines of code. On the download date, it had eight stars.

#### 3.5.5. Smart-Farm [31]

According to its developers, this project is “a DIY project for managing a farm”. This project was realized by two developers; it is not connected to the internet and, without considering the imported libraries, has 60 SloC. The Arduino is connected to a temperature–humidity sensor, an air motor, a solenoid valve, and an LCD display. On the download date, it had 17 stars.

#### 3.5.6. SmartOutlet-IOT [32]

According to its developer, this project is a “Home automation Proof of Concept system that allows the user to control a device from any client connected to the Wi-Fi network”. The Arduino receives radio-frequency commands from a Raspberry and enables or disables a relay. The Arduino is not directly connected to the internet. The part of the project related to Arduino is composed of 31 lines of code. On the download date, it had seven stars.

#### 3.5.7. IKEA PS 2014 DIY Lamp [33]

This project uses an ESP8266 to control the IKEA PS 2014 Lamp. The Arduino is connected to a stepper motor, a lamp, and the Blynk platform [34] through the internet. The project is composed of around 200 SloC. On the download date, it had five stars.

#### 3.5.8. DIY-Weather-Station [35]

As the name suggests, this project is a homemade weather station. The involved Arduino board is connected through the internet to the Blynk platform [34] and two different sensors (a DHT11 and BMP180). The first one is a temperature and humidity sensor, while the second sensor collects pressure, temperature, and altitude. Without considering the included libraries, this project has around 70 lines of code. On the download date, it had six stars.

#### 3.5.9. Oscilloscope32 [36]

This project aims to retrieve data from a smart meter (KAIFA MA105H2E AMS). The project has more than 100 lines of code and is not connected to the internet. On the download date, it had six stars.

#### 3.5.10. Mbus-Han-Kaifa [37]

The purpose of this project is to retrieve data from a smart meter (KAIFA MA105H2E AMS). The project has more than 100 lines of code, and it is not connected to the internet. On the download date, it had six stars.

#### 3.5.11. CounterStrike GlobalOffensive—Ambilight-System [38]

This project is designed to change some LEDs, reacting to the user’s in-game statistics ina PC videogame called “Counterstrike: Global Offensive”. The project contains more than 400 SloC. The Arduino fetches UDP packets from a local web server (which has to be configured by the user) and then interacts with the connected LEDs. On the download date, it had five stars.

#### 3.5.12. Regulator [39]

This project is a DIY Arduino consumption regulator built to use excess solar power for auxiliary heating. The solar power data are retrieved over SunSpec Modbus TCP. The IoT monitoring is managed through the Blynk platform [34] and a local web server. Without considering the imported libraries, the project has more than 2500 lines of code. On the download date, it had 12 stars.

#### 3.5.13. Pixel Cube [40]

This project is a DIY cube designed to track working activities. As declared by its two developers, it is strongly inspired by other tracking services such a TimeFlip [41]. Each face of the cube is associated with an activity; flipping the cube starts the time monitoring of that specific activity. The board’s orientation and time monitoring information are processed by a server-side Electron-Vue application [42]. The board is directly connected through the serial port to the machine hosting the web application. Indeed, the application does not have direct access to the internet. The code designed for running on the Arduino board is around 140 SLoC. On the download date, it had five stars.

#### 3.5.14. Control Motors with Processing [43]

The purpose of this project is to control a motor connected to an Arduino, taking advantage of a companion application (called “Processing”) with a Graphical User Interface (GUI). The machine equipped with the companion app is directly connected to the Arduino board through a serial communication. Indeed, the board does not have direct access to the internet. Without considering the imported libraries, the code running on the Arduino board comprises around 35 SLoC. On the download date, it had nine stars.

#### 3.5.15. BatteryNode [44]

This project aims to create a network of low-power IoT sensors. It is mainly composed of two classes of elements: a gateway and a remote sensor. The gateway can manage commands received both through Wi-Fi and MQTT [45] connections. Both these classes of devices are connected to Wi-Fi. The gateway could collect several types of data from the nodes, such as humidity, temperature, and pressure. Inside the repositories, many testing examples are provided. For the purpose of this analysis, we focus our attention on the previous two described components. The analyzed code is composed of around 500 SLoC. On the download date, it had 21 stars.

#### 3.5.16. Capacitive Soil Moisture Sensor [46]

According to its developer, this project is a “compact and battery-powered capacitive soil moisture sensor”. This project communicates using an NRF24L01 component (that supports 2.4 GHz radio communications). Furthermore, it is one of the very few projects to show security protection. Indeed, the application could optionally be configured to authenticate itself using an ATSHA204A [47] (a SHA-based crypto authentication device). Without considering the imported libraries, the project has around 160 SLoC. On the download date, it had 25 stars.

## 4. Results

We analyzed the repositories presented in Section 3.5 according to the classification approach described in Section 3.1, following the procedure presented in Section 3.3. We noticed that the most critical categories for the analyzed projects are reasonably the first two (i.e., Communications and Device/Services), while the Users category is the less affected one.

In the following subsections, thid paper discusses the five security categories and associates each project with related security issues. For each security issue, we describe the feasibility of an attack using one of the following labels:

1. **Feasible**: the attack could be conducted by a malicious user in certain circumstances (e.g., it can be conducted only on the local network, or a stress test should be executed to prove this issue’s feasibility).
2. **Not Feasible**: according to the described methodology, the security issue could not be exploited on the analyzed project.

It is noteworthy that the concept of “feasible” and “not feasible” are defined according to the adopted methodology, and a closer inspection (e.g., with a run-time analysis) could uncover false positives and false negatives.

### 4.1. Communications

Communication is a central aspect of the IoT, linking users and devices. From the outcomes of our analysis, we observed that the analyzed Arduino projects are very susceptible to these types of attacks. This category includes these four security issues: *Routing attacks*, *Active attacks*, *Passive attacks*, and *Flooding*.

*Routing attacks* are a type of cyber attack that targets a network’s routing protocols or infrastructure in order to disrupt or manipulate the normal flow of traffic. This class of threat can include redirecting traffic to a malicious destination, intercepting or modifying traffic, or disrupting the ability of devices to communicate with one another. *Routing attacks* can have a wide range of consequences, from temporary disruptions to more severe and long-term effects such as data breaches or service outages. For instance, attackers can conduct *Routing attacks* using ARP spoofing on a local network. This kind of attack is complicated to counter, and we did not notice any particular protection against them in any of the analyzed projects.

*Active* and *Passive attacks* can alter, modify, eavesdrop, or analyze the traffic sent or received by the Arduino. If malicious users have access to the route used by a device to reach another endpoint, they can always execute an *Active attack*, altering (and reasonably invalidating) the transmitted data. However, if transmitted data are encrypted, it is much more difficult for the attackers to conduct sophisticated attacks (because they cannot know the structure of the data). Even if encryption could be executed even at the application level, to better protect the data traffic on a local wireless network, users should at least enable a robust Wi-Fi encryption standard such as Wireless Protected Access (WPA) 2. According to the available source code, the analyzed projects never specify which kind of encryption users should adopt. For this reason, we exclude this issue from the following table, and we suppose that the Wi-Fi protocol is correctly configured with a proper standard at installation time. On the other hand, to ensure that data are safe when transferred over the internet, developers should use an encryption protocol such as Transport Layer Security (TLS) over HTTP or IPsec. No one of the analyzed projects seems to adopt such protections. However, there is a project (Section 3.5.16) that could be optionally equipped with an SHA-based crypto authentication module. If such a module is enabled, sent data are authenticated, protecting the application from active attacks.

*Flooding attacks* are a type of network attack in which an attacker attempts to flood a network or a target device with a large amount of traffic in order to overwhelm it and cause it to become unavailable to legitimate users. This can be performed by using various techniques such as ping floods, SYN floods, and UDP floods. Since many Arduino-like devices have few computational resources, they can be easily targeted by (Distributed) DoS attacks such as the previously cited ones. These attacks could even be conducted from outside the local network if the Arduino device exposes a public service such as an HTTP server. One of the easiest ways to handle this security issue is to configure a router (or a firewall) to mitigate possible attacks. In the reviewed projects, this mechanism is not mentioned. Moreover, we did not notice any kind of protection against them in the source code.

Table 2 considers how the security issues affect the considered GitHub projects.

### 4.2. Device/Services

The following four security issues belong to this category: *Physical Attacks*, *Device Subversion*, *Device Data Access*, and *Device Degradation*.

Starting with *Physical Attacks*, considering we are discussing DIY projects, these applications are not designed to work in critical scenarios. In the analyzed projects, we noticed poor error management if a component is broken or disconnected. The same issue arises when the Arduino-like board loses network connectivity. For these reasons, we consider *Physical Attacks* to be always feasible, especially if the attacker has physical access to the Arduino. Therefore, this security issue is not reported for each project in Table 2.

The *Device Subversion* issue includes attacks like device control or device capture. This kind of attack can also be conducted by having physical access to the IoT device. Considering how easy it is to physically override an Arduino-like board’s source code through its USB port, we can also view this attack as always feasible. Furthermore, controlling the Arduino can also provide access to the devices, sensors, and actuators connected to that board (directly or through the local network).

Regarding *Device Data Access*, we observed that developers often store their Wi-Fi networks’ sensitive information in plain-text strings inside these projects (i.e., the Service Set Identifier (SSID) and the associated password). Therefore, having access to the Arduino source code could often give access to the owner’s network (possibly opening the door to more severe attacks). Indeed, we discovered that storing SSID and password in plain source code is also implicitly suggested in official Arduino documentation [48]. Furthermore, considering that these projects rarely encrypt data at rest, an attacker could also steal access tokens, or the Arduino Media Access Control (MAC) addresses, to execute an Identity Spoofing attack. Moreover, when a web server is exposed, we did not notice any kind of protection against replay attacks.

To conclude, regarding *Device Degradation*, the Arduino-like boards are usually connected to the main power supply, so they are generally not affected by battery exhaustion attacks. Nevertheless, considering the low resources of a traditional Arduino board (and the lack of countermeasures in the reviewed projects), it is reasonable that the devices could fall victim to other kinds of attacks belonging to this category (like a memory exhaustion attack).

Table 3 highlight how these security issues affect the analyzed GitHub projects.

### 4.3. Users

Security threats related to Users can be divided into four categories: *Trust*, *Data Confidentiality*, *Identity Management*, and *Behavioral Threats*.

Regarding *Trust* attacks, inside our pool of applications, we did not find scenarios in which attacks such as self-promoting, good mouthing, or bad mouthing could have a place. Clearly, as we already highlighted in Section 4.2, by overriding the source code of the board, it is still possible to try to execute such attacks.

Considering *Identity Management* threats, there are usually no identities to manage in these projects; therefore, this kind of threat is rarely present in the analyzed projects.

We also found the same situation for *Data Confidentiality*. Considering that there are no identities to manage, there are almost no users’ confidential data. In only one application, we found a piece of sensitive information stored as plain text: the cellphone number of the admin users (necessary to receive notifications). If this information is stolen, on the one hand, we can have Data Confidentiality issues (such as user impersonation, identity spoofing, or phishing attack). On the other hand, an attacker could use this information, for instance, to carry out a social engineering attack (which is a Behavioral Threat).

To conclude, the Behavioral Threats category, as previously discussed, includes social engineering attacks. It is important to note that this type of attack is always a possibility when user interaction is required for the project. Additionally, since these projects typically lack authentication at the startup stage, any person in the same vicinity as the Arduino device can use it, leaving room for a potential “free riding attack”, which is another type of attack that falls under this category.

Table 4 illustrates how the described security issues can affect the considered GitHub projects. In the table, we report *only* those projects with different behavior in one of the previously cited security issues from the general description provided in this section.

### 4.4. Mobility

The security issues related to this category are the following three: *Dynamic Topology*/*Infrastructure*, *Tracking and Location Privacy*, and *Multiple Jurisdictions*.

As already specified in Section 3.3, we have conducted a source-code-level analysis of the security issues belonging to each security category. Therefore, to identify potential security vulnerabilities related to this security category, we have taken into account potential undetected changes in network topology or disconnection of connected sensors/actuators. We also considered what could happen if the network connection is lost. In addition, we even examined if the developed applications could be tracked and if the data collected or created by the application could be stored on servers under a different jurisdiction from the one in which the Arduino board will be deployed.

Starting with the *Dynamic Topology*/*Infrastructure* issues, we found one project retrieving information from a local server and a few projects interacting with other local devices. In these projects, we observed no identity verification. Therefore, in those cases, moving the device to another network could change the retrieved data and create an impact on the developed application.

Regarding *Tracking and Location Privacy* issues, there are no projects in our pool that use tracking and location functionality. Therefore, this security issue is not reported in Table 4. However, if the Arduino board is connected to the internet, it is noteworthy that an approximate location could always be retrieved from its IP address. To mitigate this issue, the developer should adopt an obfuscation mechanism (e.g., using a Virtual Private Network to access the internet).

To conclude, regarding *Multiple Jurisdictions* issues, we observed no collaborating or coordinating disjointed networks of things in DIY projects (usually, such developers create something “local”, designed to be used in their houses). However, including an external online platform inside the project (e.g., Blynk) could create this kind of issue if the deployed application is in a different country where the remote platform is hosted. In this case, following the classification described at the beginning of Section 4, we consider this issue to be relevant (feasible) if some data could be stored in different countries (e.g., cloud services), or reasonably not relevant (not feasible) otherwise.

Table 5 presents how the discussed security issues affect the analyzed GitHub projects. In this table, we used the same approach as Table 4; only relevant projects and security issues are reported.

### 4.5. Integration of Resources

In IoT, the collection, processing, storage, and usage of data are highly dependent on different infrastructures. The integration of these resources could be subject mainly to these three categories of issues: *Cross-Domain Administration*, *Cascading Resources*, and *Interoperability*.

Considering *Cross-Domain Administration* issues, we observed that the analyzed projects do not involve many decentralized nodes. Indeed, the projects are mainly designed to work on the users’ local networks. Moreover, in the analyzed projects, there are no particular policies to be configured or identities to be managed. Therefore, this security issue is not reported in Table 6.

Regarding *Cascading Resources* issues, taking control of the Arduino device could give the attacker access to that board’s data, functionalities, and capabilities. Furthermore, if the Arduino has some sensors or actuators, they can be compromised as well. Considering that some projects contain an access token to take advantage of the functionalities of the Blynk platform, after compromising the board, malicious users could retrieve that token and obtain access to the remote platform’s features.

To conclude, for *Interoperability* issues, we do not have projects with fog computing or similar functionalities, so attacking an Arduino does not affect peer systems. However, an Arduino is usually connected to many sensors, and having the possibility to control this device could give access to their information. Furthermore, we must consider again that some applications use an authentication token to contact the Blynk platform. With that token, an attacker could have access to data stored on the external platform.

Table 6 considers how the security issues affect the considered GitHub projects.

### 4.6. Results Summary

The outcome of the conducted analysis is summarized in Table 7.

## 5. Discussion

Our analysis was based on the threat model presented in [25]. This model is divided into five security categories: *Communications*, *Devices*/*Services*, *Users, Mobility*, and *Integration of Resources*. Those categories were further described in Section 3.1.

In this section, we describe some lessons learned about the behavior of hobbyist developers from the conducted analysis. Table 8 reports a quantitative summary of the observed security issues in the analyzed repositories.

To begin with, we observe that the three more relevant categories are *Communications*, *Device*/*Services*, and *Integration of Resources*. Unfortunately, the fact that *Users* and *Mobility* categories are not particularly relevant for the analyzed projects is not due to developers’ countermeasures. Indeed, most of the projects are self-contained, and often they do not manage users’ data or multiple identities. If we consider the first three cited categories, the frequency of having a feasible issue is much higher.

Proceeding the discussion category by category, the first category to analyze is *Communication*. The average frequency that an issue belonging to this category could be present in our Arduino project is 64% (the second highest value). Analyzing the Arduino-like projects, we noticed that developers usually did not encrypt data in their DIY IoT applications. However, encryption is particularly useful to avoid (or reduce) attacks belonging to this category. Indeed, encryption could significantly reduce the impact of Active and Passive attacks and mitigate some types of *Routing Attacks*. Arduino devices do not have native protection against *Flooding* attacks. According to what we read in the hobbyist programmers’ repositories, we did not find any special protection (even if we could not totally exclude the possibility that some developers configured their routers to protect the Arduino). Generally speaking, we even did not observe any kind of protection against passive attacks. Only one project could optionally support the authentication of the sent data.

Considering the *Device*/*Services* category, the average frequency at which an issue in this category is feasible is around 63%. In Section 4.2, we already highlighted how *Physical Attacks* could affect Arduino devices. Indeed, they are easy to override and break. For this reason, developers should be aware that physically protecting their devices is essential to avoid many issues belonging to this category that could be executed when a malicious user has physical access to the board (*Physical Attacks*, *Device Subversion*, and *Device Data Access*). Moreover, these boards have limited resources, so they could be an easy target for DoS attacks that could lead to *Device Degradation*. In some cases, we also noticed a couple of battery-powered boards. For these projects, even a battery-exhaustion attack could lead to *Device Degradation*. To conclude, even for this security category, encryption could mitigate some of these issues. For instance, encrypting sensitive information can increase protection against *Device Data Access* attacks. Indeed, encrypting (or at least obfuscating) sensitive information such as network SSID and password is a best practice that we did not find in the analyzed repositories.

The *Users* category is one of the two less affected categories in the analyzed projects. Starting from the first security issue, we did not notice any project in which *Trust* issues were relevant. Regarding *Data Confidentiality* issues, in our pool of projects, we rarely found applications that store users’ sensitive information. For *Identity Management* issues, we found some authentication tokens stored as plain strings in the analyzed applications. Therefore, a malicious user could steal this token and have access to the platform to which the token belongs. *Behavioral Threats* such as social engineering attacks are naturally almost always possible if a malicious user can talk with the owner of the Arduino. Moreover, there is no protection against free-riding attacks (no projects require user authentication to access the project’s functionalities).

The *Mobility* category is the other less affected category. Starting from the *Dynamic Topology*/*Infrastructure* security issues, even though we did not notice any authentication strategy to verify the identity of the connected devices, many projects were quite self-contained. For this reason, they are not subject to this class of threats. For *Tracking and Location Privacy* issues, we saw that most projects did not use geolocation data. In this way, if the application connects to the internet, an attacker could only obtain an approximate location from the public IP address. The category of *Multiple Jurisdiction* issues is generally not applicable to a DIY project developed to be used by the creators themselves. This issue could be relevant if the programmers decide to take advantage of a foreign external platform to develop an application to be used by many users.

To conclude, the last security category is *Integration of Resources*. This category has the highest average frequency of finding a security issue. This is not surprising if we consider that Arduino-like boards are often logically connected to other endpoints or physically connected to sensors/actuators. However, considering that these connections rarely involve many decentralized nodes, and having *Cross Domain Administration* issues is reasonably uncommon. Conversely, *Cascading Resources* attacks are generally quite feasible. Indeed, once the board is compromised, there are generally no authentication mechanisms between the board and the connected resources. Moreover, some applications use an authentication token to obtain access to remote platforms’ functionalities. Since authentication tokens are often hard-coded in the source code of the applications, they can be exploited not only for cascading resources but also to have *Interoperability* issues. Indeed, by stealing this token, malicious users can have the possibility of executing some actions in place of the actual token’s owner. Furthermore, when the board takes advantage of functionalities offered by other endpoints available in the same network, there is no need to steal such a token. Therefore, compromising the board could offer a potential attacker the capability of exploiting the services offered by these endpoints with negligible additional effort.

## 6. Conclusions

In this research, we have conducted a source-code level analysis of security issues in DIY Arduino-like projects reasonably developed by hobbyist programmers. Our study is focused on the source code that an Arduino-like device can execute.

Analyzing those repositories shows that novice developers rarely pay attention to cybersecurity concepts. Therefore, we highlighted how many kinds of attacks are feasible in the evaluated projects. Considering that these projects are freely available on the internet, other users could download and install them on their own boards without considering the potential security issues. Furthermore, novice developers could use these projects as practical examples to develop their own. In this way, newly developed projects may take these security issues with them. After this analysis, we can conclude that users should not install this kind of DIY project lightly, especially if any (more crucial) systems are in the same network.

In conclusion, this research is a first step towards a better understanding of security issues in Arduino-like projects, and we hope that it will encourage further research in this area. We believe that identifying potential security weaknesses in novice programmers’ IoT projects can lead to the development of more secure systems in the future.

### Future Work

As the number of IoT devices and projects continues to grow, it is crucial that we continue to research and develop new methods for protecting these devices from potential threats and support inexperienced programmers in their projects. Moving forward, we plan to compare our findings with a set of static checkers. The comparison between our findings and the outcome of these tools could even lead to the creation of a new static checker that can detect security issues in Arduino-like projects. Additionally, we also plan to observe which issues can be found through dynamic analysis. To conclude, we want to try to estimate the severity in case a threat is exploited by an attacker in a smart home setup.

## Figures and Tables

**Table 1:** Summary of the projects involved in the security analysis.

ID	GitHub Project Name	Stars	SLoC	Contributors	Internet Access
01	iotinator	8	~2000	1	Yes
02	Probee	6	~960	1	No
03	Arduino Commands	7	~300	1	Yes
04	TwitterMoodLight	8	~760	1	Yes
05	Smart-Farm	17	~60	2	No
06	SmartOutlet-IOT	7	~30	1	No
07	IKEA PS 2014 DIY Lamp	5	~200	1	Yes
08	DIY-Weather-Station	6	~70	1	Yes
09	Oscilloscope32	6	~100	1	No
10	Mbus-han-kaifa	6	~100	1	No
11	CounterStrike GlobalOffensive—Ambilight-System	5	~400	1	Yes
12	Regulator	12	~2500	1	Yes
13	Pixel Cube	5	~140	2	No
14	Control-Motors-with-Processing-	9	~35	1	No
15	BatteryNode	21	~500	1	Yes
16	Capacitive Soil Moisture Sensor	25	~160	1	No

**Table 2:** “Communications” security issues inside the analyzed projects.

Github Project	Routing Attacks	Active Attacks	Passive Attacks	Flooding
**iotinator**	**Feasible**: e.g., ARP spoofing.	**Feasible**: no data encryption in transmission.	**Feasible**: no data encryption in transmission.	**Feasible**: there is no declared protection.
**Probee**	**Not Feasible**: no network connection.	**Not Feasible**: no data transmission.	**Not Feasible**: no data transmission.	**Not Feasible**: no network connection.
**Arduino ** **Commands**	**Feasible**: e.g., ARP spoofing.	**Feasible**: connection to a local HTTP server without encryption.	**Feasible**: connection to a local HTTP server without encryption.	**Feasible**: there is no declared protection.
**TwitterMoodlight**	**Feasible**: e.g., ARP spoofing.	**Feasible**: no data encryption in transmission.	**Feasible**: no data encryption in transmission.	**Feasible**: there is no declared protection.
**Smart-Farm**	**Not Feasible**: no network connection.	**Not Feasible**: no data transmission.	**Not Feasible**: no data transmission.	**Not Feasible**: no network connection.
**SmartOutlet-IOT**	**Feasible**: no network connection, the attack has to be conducted through RF connection.	**Feasible**: received data are not encrypted.	**Feasible**: received data are not encrypted.	**Feasible**: there is no declared protection.
**IKEA PS 2014 DIY Lamp**	**Feasible**: e.g., ARP spoofing.	**Feasible**: communication with Blynk platform is not encrypted.	**Feasible**: communication with Blynk platform is not encrypted.	**Feasible**: there is no declared protection.
**DIY-Weather-Station**	**Feasible**: e.g., ARP spoofing.	**Feasible**: Communication with Blynk is not encrypted.	**Feasible**: Communication with Blynk is not encrypted.	**Feasible**: there is no declared protection.
**Oscilloscope32**	**Not Feasible**: no network connection.	**Not Feasible**: no data transmission.	**Not Feasible**: no data transmission.	**Not Feasible**: no network connection.
**mbus-han-kaifa**	**Feasible**: e.g., ARP spoofing.	**Not Feasible**: no data transmission.	**Not Feasible**: no data transmission.	**Feasible**: there is no declared protection.
**CounterStrike ** **GlobalOffensive—Ambilight-System**	**Feasible**: e.g., ARP spoofing.	**Feasible**: connection to a local HTTP server without encryption.	**Feasible**: connection to a local HTTP server without encryption.	**Feasible**: there is no declared protection.
**Regulator**	**Feasible**: e.g., ARP spoofing.	**Feasible**: it uses Telnet; communication with Blynk is not encrypted.	**Feasible**: it uses Telnet; communication with Blynk is not encrypted.	**Feasible**: there is no declared protection.
**Pixel Cube**	**Not Feasible**: Arduino directly connected through serial port.	**Not Feasible**: Arduino directly connected through serial port.	**Not Feasible**: Arduino directly connected through serial port.	**Feasible**: there is no declared protection. The connected machine could be exploited to flood the board.
**Control-Motors-with-Processing-**	**Not Feasible**: Arduino is directly connected through serial port.	**Not Feasible**: Arduino is directly connected through serial port.	**Not Feasible**: Arduino is directly connected through serial port.	**Feasible**: there is no declared protection. The connected machine could be exploited to flood the board.
**BatteryNode**	**Feasible**: e.g., ARP spoofing.	**Feasible**: received data are not encrypted.	**Feasible**: received data are not encrypted.	**Feasible**: there is no declared protection.
**Capacitive Soil Moisture Sensor**	**Feasible**: no network connection, the attack has to be conducted through RF connection.	**Not Feasible**: If the crypto authentication device is enabled, messages cannot be altered (otherwise Feasible).	**Feasible**: ATSHA204A provides authentication, not confidentiality (i.e., encryption).	**Not Feasible**: data are never read, only sent.

**Table 3:** “Device/Services” security issues inside the analyzed projects.

Github Project	Device Data Access	Device Degradation
**iotinator**	**Feasible**: access to sensitive information on the board (such as Wi-Fi credentials).	**Feasible**: e.g., using DoS attacks (such as SYN flooding).
**Probee**	**Feasible**: access to sensitive information on the board (Wi-Fi credentials are stored for compile time).	**Not Feasible**: Arduino not connected to the network.
**Arduino Commands**	**Feasible**: access to sensitive information on the board (such as Wi-Fi credentials).	**Feasible**: e.g., using DoS attacks (such as SYN flooding).
**TwitterMoodlight**	**Feasible**: access to sensitive information on the board (such as Wi-Fi credentials).	**Not Feasible**: Arduino fetches only data from Twitter.
**Smart-Farm**	**Feasible**: it is possible to get the data of the associated sensors.	**Not Feasible**: Arduino not connected to the network.
**SmartOutlet-IOT**	**Not Feasible**: no sensitive data.	**Feasible**: through Radio Frequency messages [49].
**IKEA PS 2014 DIY Lamp**	**Feasible**: access to sensitive information on the board (such as Wi-Fi credentials or Blynk token).	**Feasible**: the Arduino or motor could be broken.
**DIY-** **Weather-Station**	**Feasible**: access to sensitive information on the board (such as Wi-Fi credentials or Blynk token).	**Not Feasible**: device “does not listen” for incoming messages.
**Oscilloscope32**	**Not Feasible**: no sensitive data.	**Not Feasible**: no network connection.
**mbus-han-kaifa**	**Feasible**: access to sensitive information on the board (such as Wi-Fi credentials).	**Not Feasible**: device “does not listen” for incoming messages.
**CounterStrike ** **GlobalOffensive—Ambilight-System**	**Feasible**: access to sensitive information on the board (such as Wi-Fi credentials).	**Feasible**: e.g., using DoS attacks (such as SYN flooding).
**Regulator**	**Feasible**: access to sensitive information on the board (such as Wi-Fi credentials or Blynk tokens).	**Feasible**: the Arduino or the sensors/actuators could be broken.
**Pixel Cube**	**Not Feasible**: no sensitive data.	**Feasible**: e.g., using DoS attacks (such as SYN flooding).
**Control-Motors-with-Processing-**	**Not Feasible**: no sensitive data.	**Feasible**: e.g., using DoS attacks (such as SYN flooding).
**BatteryNode**	**Feasible**: access to sensitive information on the boards (such as Wi-Fi credentials).	**Feasible**: e.g., using DoS attacks (such as SYN flooding).
**Capacitive Soil Moisture Sensor**	**Not Feasible**: no sensitive data.	**Not Feasible**: device “does not listen” for incoming messages.

**Table 4:** “Users” security issues in the analyzed projects.

Github Project	Data Confidentiality	Identity Management	Behavioral Threats
**iotinator**	**Feasible**: Phone numbers stored in clear on the Arduino.	**Not Feasible**: actually, all users are admins.	**Feasible**: Social Engineering and Free Riding attacks could always be feasible for the analyzed projects.
**Probee**	**Not Feasible**: no sensitive data.	**Not Feasible**: no users.	**Not Feasible**: once started, there is no user interaction.
**DIY-** **Weather-Station**	**Not Feasible**: no sensitive data.	**Feasible**: stolen Blynk access token could be used to authenticate other devices.	**Not Feasible**: application reads data from sensors, only.
**IKEA PS 2014 DIY Lamp**	**Not Feasible**: no sensitive data.	**Feasible**: stolen Blynk access tokens could be used to authenticate other devices.	**Feasible**: Social Engineering and Free Riding attacks could always be feasible for the analyzed projects.
**Regulator**	**Not Feasible**: no sensitive data.	**Feasible**: stolen Blynk access tokens could be used to authenticate other devices.	**Feasible**: Social Engineering and Free Riding attacks could always be feasible for the analyzed projects.
**Capacitive Soil Moisture Sensor**	**Not Feasible**: no sensitive data.	**Not Feasible**: no users.	**Not Feasible**: once started, there is no user interaction.

**Table 5:** “Mobility” security issues inside the analyzed projects.

Github Project	Dynamic Topology/Infrastructure	Multiple Jurisdictions
**Probee**	**Feasible**: the Arduino has to interact with a Raspberry.	**Not Feasible**: no network connection.
**Arduino commands**	Not Feasible: no issues if local topology changes.	**Not Feasible**
**TwitterMoodlight**	**Not Feasible**: no issues if local topology changes.	**Feasible**: data retrieved from Twitter could be relevant for data privacy.
**Smart-Farm**	**Not Feasible**: no issues if local topology changes.	**Not Feasible**
**SmartOutlet-IOT**	**Feasible**: it accepts radio frequency commands from anyone.	**Not Feasible**
**IKEA PS 2014 DIY Lamp**	**Not Feasible**: no issues if local topology changes.	**Feasible**: relying on Blynk platform could be relevant for data privacy.
**DIY-** **Weather-Station**	**Not Feasible**: no issues if local topology changes.	**Feasible**: relying on Blynk platform could be relevant for data privacy.
**Oscilloscope32**	**Not Feasible**: no issues if local topology changes.	**Not Feasible**
**Mbus-han-kaifa**	**Not Feasible**: no issues if local topology changes.	**Not Feasible**
**CounterStrike ** **GlobalOffensive—Ambilight-System**	**Feasible**: the Arduino retrieves data from a local server.	**Not Feasible**
**Regulator**	**Feasible**: the Arduino interacts with Modbus TCP (no authentication).	**Feasible**: relying on Blynk platform could be relevant for data privacy.
**Pixel Cube**	**Feasible**: the Arduino interacts with another machine through serial port (no authentication).	**Not Feasible**
**Control-Motors-with-Processing-**	**Feasible**: the Arduino interacts with another machine through serial port (no authentication).	**Not Feasible**
**BatteryNode**	**Feasible**: the project is a network of devices that relies on a gateway.	**Not Feasible**
**Capacitive Soil Moisture Sensor**	**Not Feasible**: no issues if local topology changes.	**Not Feasible**

**Table 6:** Integration of Resources security issues inside the analyzed projects.

Github Project	Cascading Resources	Interoperability
**iotinator**	**Feasible**: it is possible to have access to the website.	**Feasible**: it is possible to have access to website’s data.
**Probee**	**Feasible**: controlling the connected devices/sensors/actuators.	**Not Feasible**: no interaction with other resources.
**Arduino Commands**	**Feasible**: controlling the connected devices/sensors/actuators.	**Not Feasible**: no peer resources, no available data.
**TwitterMoodlight**	**Feasible**: controlling the connected devices/sensors/actuators.	**Not Feasible**: no peer resources, no available data.
**Smart-Farm**	**Feasible**: controlling the connected devices/sensors/actuators.	**Not Feasible**: no interaction with other resources.
**SmartOutlet-IOT**	**Not Feasible**: read-only.	**Not Feasible**: no interaction with other resources.
**IKEA PS 2014 DIY Lamp**	**Feasible**: Blynk platform auth token.	**Feasible**: Blynk platform auth token.
**DIY-** **Weather-Station**	**Feasible**: Blynk platform auth token.	**Feasible**: Blynk platform auth token.
**Oscilloscope32**	**Feasible**: controlling the connected display.	**Not Feasible**: no peer resources, no available data.
**mbus-han-kaifa**	**Feasible**: changing connected meter threshold values.	**Feasible**: reading meter values.
**CounterStrike ** **GlobalOffensive—Ambilight-System**	**Feasible**: controlling the connected devices/sensors/actuators.	**Feasible**: obtaining data from the HTTP server.
**Regulator**	**Feasible**: Blynk platform auth token.	**Feasible**: Blynk platform auth token.
**Pixel Cube**	**Feasible**: controlling the connected devices/sensors/actuators.	**Feasible**: altering the normal working flow of the connected application.
**Control-Motors-with-Processing-**	**Feasible**: controlling the connected devices/sensors/actuators.	**Not Feasible**: read only.
**BatteryNode**	**Feasible**: controlling the connected devices/sensors/actuators.	**Feasible**: controlling the gateway it is possible to override the source code of the connected nodes Over-the-Air (OTA).
**Capacitive Soil Moisture Sensor**	**Feasible**: controlling the connected devices/sensors/actuators.	**Feasible**: controlling the board it will be possible to send altered information through RF module.

**Table 7:** Summary of the security issues potentially present in each project divided by category.

ID	GitHub Project Name	Comm.	Device/Services	Users	Mobility	Int. of Resources	Total
01	iotinator	4/4	2/2	2/3	0/2	2/2	10/13
02	Probee	0/4	1/2	0/3	1/2	1/2	3/13
03	Arduino Commands	4/4	2/2	1/3	0/2	1/2	8/13
04	TwitterMoodLight	4/4	1/2	1/3	1/2	1/2	8/13
05	Smart-Farm	0/4	1/2	1/3	0/2	1/2	3/13
06	SmartOutlet-IOT	4/4	1/2	1/3	1/2	0/2	7/13
07	IKEA PS 2014 DIY Lamp	4/4	2/2	2/3	1/2	2/2	11/13
08	DIY-Weather-Station	4/4	1/2	1/3	1/2	2/2	9/13
09	Oscilloscope32	0/4	2/4	1/3	0/2	1/2	4/13
10	mbus-han-kaifa	2/4	1/2	1/3	0/2	2/2	6/13
11	CounterStrike GlobalOffensive—Ambilight-System	4/4	2/2	1/3	1/2	2/2	10/13
12	Regulator	4/4	2/2	2/3	2/2	2/2	12/13
13	Pixel Cube	1/4	1/2	1/3	1/2	2/2	6/13
14	Control-Motors-with-Processing-	1/4	1/2	1/3	1/2	1/2	5/13
15	BatteryNode	4/4	2/2	1/3	1/2	2/2	10/13
16	Capacitive Soil Moisture Sensor	2/4	0/2	0/3	0/2	2/2	4/13

**Table 8:** Summary of the security issues observed in the analyzed repositories. The table does not consider those security issues not reported in the other tables.

Security Category	Feasibility for Each Category	Security Issue	Issue Not Feasible	Issue Feasible
Communications	64%	Routing Attack	5 (31%)	11 (69%)
		Active Attack	7 (44%)	9 (56%)
		Passive Attack	6 (37%)	10 (63%)
		Flooding	5 (31%)	11 (69%)
Device/Services	63%	Device Data Access	5 (31%)	11 (69%)
		Device Degradation	7 (44%)	9 (56%)
Users	35%	Data Confidentiality	15 (94%)	1 (6%)
		Identity Management	13 (81%)	3 (19%)
		Behavioral Threats	3 (19%)	13 (81%)
Mobility	34%	Dynamic Topology/Infrastructure	9 (56%)	7 (44%)
		Multiple Jurisdiction	12 (75%)	4 (25%)
Integration of Resources	75%	Cascading Resources	1 (6%)	15 (94%)
		Interoperability	7 (44%)	9 (56%)

## Data Availability

When this paper was submitted, all the analyzed repositories were freely available on GitHub. All the links are reported in the references.

## Abbreviations

ARP	Address Resolution Protocol
COTS	Commercial Off-the-Shelf
DDoS	Distributed Denial of Service
DoS	Denial of Service
DIY	Do It Yourself
GHz	gigahertz
GUI	Graphical User Interface
ICT	Information and Communication Technologies
IDE	Integrated Development Environment
IoT	Internet of Things
IP	Internet Protocol
IPsec	IP Security
HTTP	Hypertext Transfer Protocol
HTTPS	HTTP Secure
LCD	Liquid Crystal Display
LED	Light Emitting Diode
MAC	Media Access Control
MQTT	Message Queuing Telemetry Transport
OTA	Over-the-Air
RF	Radio Frequency
SHA	Secure Hash Algorithm
SLoC	Source Lines of Code
SSID	Service Set Identifier
TCP	Transmission Control Protocol
TLS	Transport Layer Security
UDP	User Datagram Protocol
WPA	Wireless Protected Access
